# Use of Virtual Technology in Teaching Human Anatomy in Medical Schools: A Systematic Review

**DOI:** 10.7759/cureus.97969

**Published:** 2025-11-27

**Authors:** Abdelhadi Elsayed, Eram Yousif, Khalid H Mohamed, Saqib Samad, Mohamed A Abdelrahim

**Affiliations:** 1 Internal Medicine, University Hospitals Dorset NHS Foundation Trust, Poole, GBR; 2 Anatomical Sciences, St. George's University School of Medicine, St. George, GRD; 3 Neurology, Sheffield Teaching Hospitals NHS Foundation Trust, Sheffield, GBR; 4 Trauma and Orthopedics, University Hospitals Dorset NHS Foundation Trust, Poole, GBR

**Keywords:** anatomy education, augmented reality, medical students, mixed reality, systematic review, three-dimensional visualization, virtual reality

## Abstract

Human anatomy has traditionally been taught through cadaveric dissection and textbooks. Recent advances in three-dimensional (3D) visualization, virtual reality (VR), and augmented reality (AR) have introduced interactive approaches that may enhance spatial understanding. This systematic review evaluated the effectiveness of these virtual technologies compared with conventional anatomy teaching. Following PRISMA 2020 guidelines, 27 randomized controlled trials were identified that compared 3D visualization, immersive VR, AR, mixed reality (MR), and simulator systems with traditional resources such as cadaveric dissection, atlases, and 2D images. Overall, virtual technologies achieved comparable or superior learning outcomes, with the most significant benefits observed in complex spatial topics, including neuroanatomy, cardiac anatomy, and the middle and inner ear. Immersive and interactive 3D tools significantly improved conceptual understanding and examination performance, while MR achieved results equivalent to cadaveric dissection with approximately 40% less teaching time. Students with lower visual-spatial ability benefited particularly from stereoscopic 3D displays. Across all modalities, virtual technologies consistently outperformed traditional methods in subjective outcomes, including motivation, engagement, and perceived spatial clarity. However, the included trials predominantly assessed knowledge-based and subjective learning outcomes and did not evaluate the hands-on practical skills uniquely supported by cadaveric dissection. These findings indicate that advanced virtual technologies represent an effective, efficient, and inclusive complement to traditional anatomy teaching and may play a key role in modernizing medical education. This review involved analysis of published data only and did not require ethical approval.

## Introduction and background

Human anatomy remains a foundational subject in medical education, traditionally delivered through established pedagogical approaches such as cadaveric dissection and textbook-based learning. These methods have long been central to developing a comprehensive understanding of the human body. However, the landscape of medical education is continually evolving with advancements in technology. The advent of virtual technologies, including three-dimensional (3D) imaging, virtual reality (VR), and augmented reality (AR), has introduced innovative teaching modalities [1]. These technologies offer new ways to enhance the learning experience by presenting complex anatomical structures in immersive, interactive formats [1].

Despite the growing adoption of these virtual tools in health sciences education, their precise benefits and overall effectiveness in student learning, particularly in anatomy, remain a subject of ongoing investigation and, at times, remain unclear [1]. There is a recognized need to systematically evaluate how these modern approaches compare to traditional teaching methods in terms of educational outcomes [2]. This systematic review aims to address this critical gap by rigorously assessing the current evidence on the use of virtual technology (VT) in teaching human anatomy in medical schools.

## Review

Methodology

Study Design and Protocol

This systematic review adhered to the Preferred Reporting Items for Systematic Reviews and Meta-Analyses (PRISMA 2020) guidelines. A predefined protocol was established before data collection commenced. The primary aim was to evaluate the effectiveness of various 3D and technology-enhanced teaching modalities (including VR, AR, mixed reality (MR), and other digital 3D visualizations) on anatomy learning outcomes among medical and allied health students. The research question guiding this review was: How do 3D and technology-enhanced learning modalities compare with traditional anatomy teaching methods in improving learners’ understanding, engagement, and satisfaction?

Search Strategy

A comprehensive electronic literature search was performed across multiple databases, including PubMed, MEDLINE, Cochrane Library, and PsycINFO, covering the period from January 2000 to June 2025. The search strategy involved combinations of keywords and MeSH terms related to anatomy education and visualization technologies, such as (“anatomy education” OR “anatomical teaching”) AND (“three-dimensional” OR “3D” OR “virtual reality” OR “augmented reality” OR “mixed reality” OR “digital visualization” OR “simulation”). Additionally, reference lists of included studies and prior reviews were manually screened to identify further eligible publications.

Inclusion and Exclusion Criteria

The inclusion criteria specified that only randomized controlled trials (RCTs) were considered. Eligible participants were medical students at any level of study. The interventions used virtual technologies such as 3D images, VR, AR, and MR in teaching human anatomy. These were compared with traditional teaching methods, including cadaveric dissection, textbooks, atlases, or 2D images. The outcomes assessed included knowledge retention, examination performance, student satisfaction, and engagement.

The exclusion criteria ruled out non-comparative or descriptive studies, reviews, conference abstracts, and editorials. Studies focusing on surgical simulation rather than basic human anatomy education were also excluded, as well as those lacking quantitative or qualitative outcome data.

Screening and Selection Process

All retrieved records were managed for de-duplication. Two reviewers independently screened titles and abstracts for relevance. Studies deemed potentially eligible were retrieved in full text for further assessment. Discrepancies were resolved through discussion and consensus. The PRISMA flow diagram summarizes the screening process, detailing the number of studies excluded and the reasons for their exclusion.

Data Extraction

A standardized data extraction form was used to gather essential information from each included study. This information covered the author(s), year, and country of publication; the study design and sample size; and the type of intervention applied, such as VR, AR, MR, or 3D models. It also recorded the comparator used, whether traditional teaching methods, textbooks, cadaveric dissection, or 2D digital resources. The participant level (undergraduate or postgraduate) was noted, along with the learning outcomes measured, including knowledge tests, spatial understanding, and satisfaction. The main findings and any reported limitations were also documented.

Quality Assessment and Risk of Bias

The Bias 2 tool (RoB-2; Cochrane, London, UK) was used to assess RCTs [3]. Each domain was rated as low risk, some concerns, or high risk. An abbreviated RoB-2 rating (overall judgment per study) was included in the summary table for concise reporting. This quality assessment informed the interpretation of findings within the narrative synthesis.

Data Synthesis

Because of the variability in study designs, interventions, and outcome measures, conducting a meta-analysis was not feasible. Therefore, a qualitative (narrative) synthesis was performed instead. The studies were thematically grouped into categories focusing on knowledge acquisition and test performance, spatial understanding, learner engagement and satisfaction, and practicality and implementation factors. Patterns and trends were then summarized to highlight consistencies, contradictions, and quality-related differences across the studies.

Ethical Considerations

This study did not involve primary data collection and thus did not require ethical approval. All included studies were publicly available and adequately referenced.

Results

Study Characteristics

**Figure 1:**
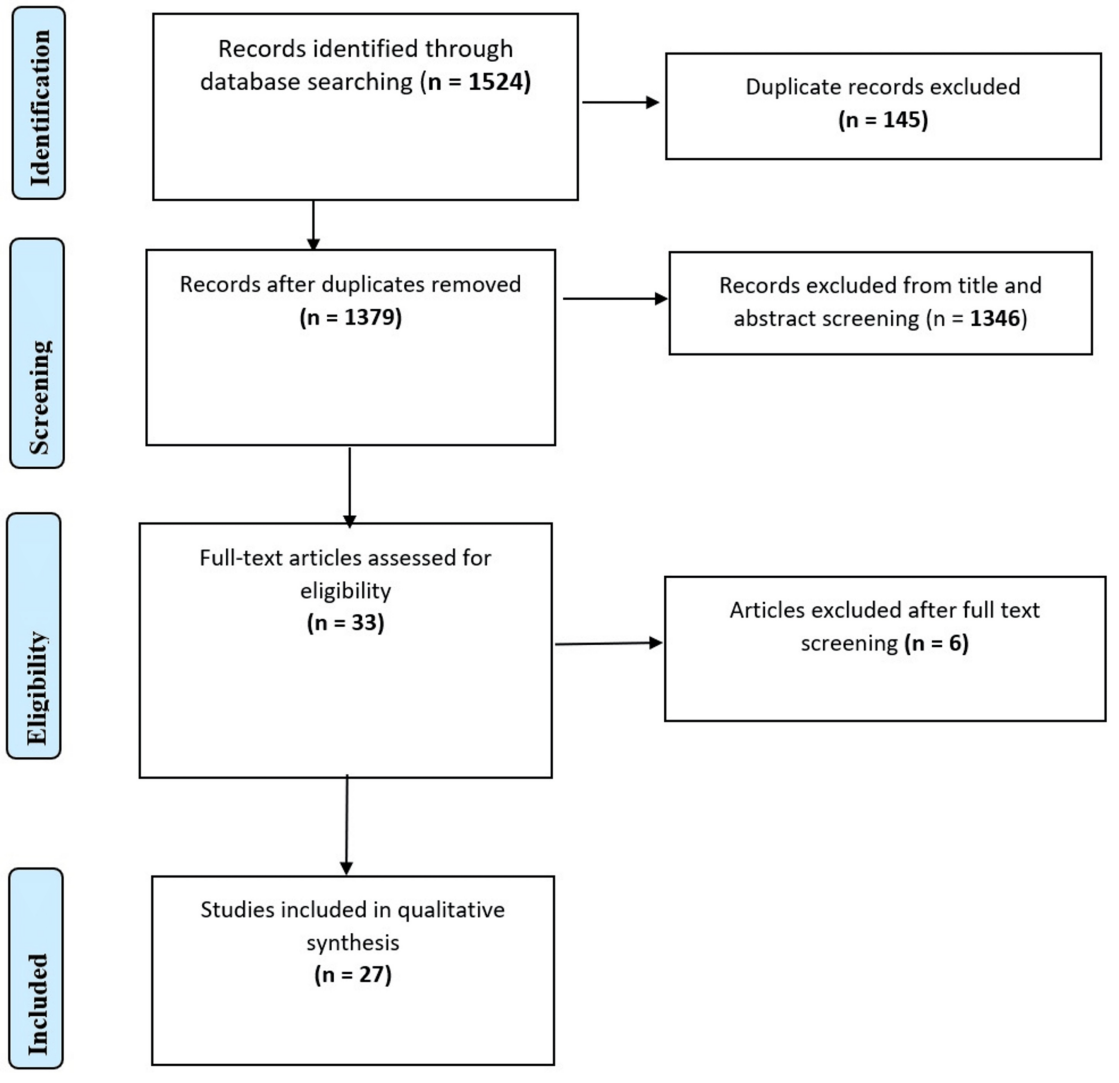
PRISMA flowchart PRISMA: Preferred Reporting Items for Systematic Reviews and Meta-Analyses

This systematic review includes 27 RCTs that evaluated the impact of various forms of VT on anatomy learning among medical students. The studies encompassed participants across all levels of medical education, from preclinical to final-year learners [4,5]. The study selection process is summarized in the PRISMA 2020 flowchart (Figure 1) below, which outlines the identification, screening, eligibility assessment, and inclusion of studies in this review.

Interventions met the eligibility criteria of involving VT (3D images, AR, or MR) in teaching human anatomy. These interventions represented a spectrum of immersion and interactivity.

Immersive VR includes studies using head-mounted displays (HMDs) or high-fidelity simulators designed to create fully immersive 3D environments, typically allowing navigation, manipulation, and interaction that goes beyond a standard screen display [4,6-14]. AR and MR include technologies explicitly identified as AR, MR, or holographic (e.g., HoloLens) [15-19]. 3D visualization includes screen-based interactive models (VI3DM), 3D PDFs, and stereoscopic projection systems that do not use HMDs [5,20-30].

Comparators were traditional teaching methods, ranging from high-fidelity physical methods, such as cadaveric dissection [18], gross prosections [27], or cadaveric bone models [12], to conventional 2D resources, including anatomical atlases, textbooks, static 2D images, and PowerPoint slides [5,9].

Anatomical structures studied predominantly involved regions requiring advanced spatial reasoning: the intricate anatomy of the middle and inner ear [16,24], neuroanatomy tracts and ventricles [8,10], complex cardiac and coronary anatomy [4,11], and musculoskeletal/skeletal structures [15,30].

Risk of Bias Assessment

The methodological quality of the included RCTs was assessed using the RoB-2 tool. Of the 27 studies, 12 were rated as "low risk," indicating robust design, such as blinded assessment of evaluation results and low attrition [11,24].

Eleven studies were categorized as "some concerns," often due to the inherent difficulty of blinding participants to the intervention (e.g., participants knew whether they were wearing a VR headset or reading a textbook) [10,16]. Other issues included reliance on subjective measures (e.g., satisfaction surveys) or a lack of standardization, such as retention tests conducted without proctoring [12].

Four studies were rated as "high risk of bias." This rating typically stemmed from high rates of missing outcome data (attrition bias) for retention assessments [13,19] or extremely small sample sizes [6]. For instance, one cluster-randomized trial experienced severe attrition, with only 34 of 85 trained students completing the examination [25].

All extracted data were organized into a summary table (Table 1) for narrative synthesis.

**Table 1:** Summary table of the included studies AG: anatomy atlas group (2D anatomical atlas), AR: augmented reality, CAs: coronary angiograms, CG: control group, CM: computer-based module (web-based, static 3D images), CT: computed tomography, DL: desktop learning group (static images via slides), DICOM: digital imaging and communications in medicine, 3D: three-dimensional, 3DG: three-dimensional human anatomy application group, 3DP: three-dimensional printed (physical) model, 3DV: three-dimensional virtual model, HMD: head-mounted display, HG: HoloLens group (Microsoft HoloLens mixed-reality group), IMMS: instructional materials motivation survey, IVR: immersive virtual reality, MG: model group (human anatomy models), MP: multiplayer, MR: mixed reality, PPT: PowerPoint presentation, RCT: randomized controlled trial, RoB: risk of bias, SIM: simulator (high-fidelity simulation system), S3D: stereoscopic three-dimensional, SP: single player, VR: virtual reality, VHD: visible human dataset (software), VI3DM: virtual interactive 3D model (interactive 3D screen-based model), VRML: virtual reality modeling language, MCQs: multiple-choice questions, RoB: risk of bias

Study ID/first author (year)	Study design/sample size	Anatomical structure/region studied	Intervention (level of immersion/interactivity)	Comparator (level of immersion/interactivity)	Outcomes (knowledge, satisfaction, etc.)	Key results/findings	RoB assessment
Chauhan (2024) [30]	RCT (parallel group)/n=200	Multiple structures (e.g., liver, humerus, thoracic vertebra)	VI3DM (interactive 3D models displayed on a screen-based application, allowing manipulation and interaction)	Conventional (2D photographs)	Knowledge acquisition/conceptualisation	Experimental group scores improved significantly in the post-test for all five anatomical specimens (p<0.05)	Low risk
Torquato (2023) [14]	Experimental RCT (crossover design)/n=42	Upper limb, lower limb, spine/back, head/neck	VR (immersive sessions, interactive tool, supplemental use)	Conventional materials (implied traditional methods: lectures, textbooks, plastic models, and a few cadaveric specimens)	Knowledge retention (short, medium, long term), motivation/perception	No significant differences were found in knowledge retention (short-, medium-, or long-term). Participants’ motivation and perceptions were overall positive	Some concerns
Chauhan (2023) [20]	RCT (parallel group)/n=200	Sacrum	VI3DM (interactive 3D models on screen-based platform, annotated, manipulated)	Conventional (2D images/teaching aid) + access to physical sacrum specimen	Conceptualization/knowledge (external features, relations, attachments)	VI3DM effectively increased the total post-test score compared to the conventional teaching aid (p<0.005)	Low risk
Veer (2022) [19]	RCT/n=67	Lungs and heart (asthma/interdisciplinary concepts)	MR HoloLens (interactive 3D model, voice/hand commands, dissecting layers, manipulation)	Textbook-style (printed pamphlet with text and 2D screenshots/images)	Learning (post-test scores), knowledge retention (2 weeks), experience/perceptions	Textbook-style resource resulted in significantly higher post-test scores than the MR group (p=0.011). No difference in knowledge retention scores. MR is perceived as more favourable and enjoyable	High risk
Copson (2021) [13]	RCT/n=47	Temporal bone anatomy (clinically oriented)	VR module (M3D/S3D) (Interactive 3D model, virtual dissection using haptic device, rotation, zoom; S3D adds stereoscopic depth)	PPT (2D, non-interactive PowerPoint presentation, screenshots + audio)	Knowledge acquisition (identification, structural relations, clinical relevance), attitudes/satisfaction	3D technologies showed significant benefit in structural relations and clinical relevance outcomes compared to baseline. S3D had greater user satisfaction, perceived effectiveness, and ease of use	High risk
Stojanovska (2019) [18]	Prospective RCT/n=64	Musculoskeletal anatomy (upper and lower limb)	MR (Microsoft HoloLens, group course, required 3.6 h curricular time)	Traditional (cadaveric dissection, required 6 h curricular time)	Practical examination scores (MR vs. cadaver exam)	No statistical difference between MR practical exam scores and cadaver exam scores (p>0.05). MR was more efficient (requiring less curricular time)	Low risk
Koucheki (2022) [12]	RCT (crossover noninferiority)/n=50 enrolled, 36 completed	Skeletal anatomy (upper and lower limbs)	IVR (HMD/VR headsets) allows manipulation of virtual models	Cadaveric bone models (direct physical models)	Performance on pre-, post-, and retention tests, satisfaction, and usability	IVR and cadaveric bone are equally effective in skeletal anatomy education. IVR is rated highly valuable for 3D orientation and relationships	Some concerns
Weeks (2020) [17]	Randomized-controlled study/n=30	Head and neck anatomy-radiology correlation	AR headset (3D CT hologram, stereoscopic depth cues, interactive viewing)	Screen-based visualization (navigable 2D slices/orientations on a laptop screen)	Examination performance (short-term recall), perceived understanding, subjective feedback	AR group showed significantly superior performance on the post-test compared to the screen group (p = 0.02). Both methods were highly popular	Some concerns
Gnanasegaram (2020) [16]	Prospective RCT/n=29	Middle and inner ear anatomy	HG (Microsoft HoloLens HMD, 3D visualisation, observing/walking around the model, spatial exploration)	DL (static 3D images via slides) or web-based CM (same static images on computers)	Anatomic knowledge post-intervention, satisfaction, engagement, and spatial relationships	Knowledge improvement was equal across all groups. HG was rated significantly higher than the other groups on overall effectiveness, spatial relationships, engagement, and motivation (p<0.001)	Some concerns
Maresky (2019) [4]	Prospective RCT (pilot study)/n=42	Cardiac anatomy (heart)	VR simulation (30-minute immersive visual–spatial environment, the learner can interact three-dimensionally)	Independent study (control group, unstructured learning)	Examination performance (conventional and visual–spatial content), effectiveness	VR group scored 23.9% higher overall (p<0.001) than the control group. VR found to be an effective tool	Some concerns
Fischer (2018) [11]	Prospective RCT/n=118	Coronary anatomy, angiography projections, and real CAs interpretation	SIM (high-fidelity simulator, allows individual manipulation, 3D view switching)	Traditional (PowerPoint-based course, face-to-face teaching)	Knowledge acquisition, competencies, and student satisfaction	SIM students had significantly higher global scores (p<0.001). Student satisfaction was significantly higher in the SIM group (98% vs. 75%, p<0.001)	Low risk
Stepan (2017) [10]	RCT/n=66	Neuroanatomy (ventricular system, cerebral vasculature)	Immersive VR interactive model (Oculus Rift HMD, 3D video + free interaction with controller, tracked head movements)	Online textbooks (Web-based, text, and 2D images)	Anatomy knowledge (post, retention quizzes), engagement, and motivation (IMMS)	No significant difference in anatomy knowledge (post-test or retention). VR group reported significantly higher motivation, engagement, enjoyment, and usefulness (p<0.01)	Some concerns
Agbetoba (2017) [29]	Multi-institutional RCT (crossover)/n=65	Frontal recess and frontal sinus drainage pathway anatomy	3D learning (virtual planning software, dynamic 3D perspective, viewing reconstructed images)	Traditional 2D (standard DICOM viewing software, conventional sagittal/coronal images, review of printed article)	Self-assessment of knowledge/understanding, perceived effectiveness, and spatial orientation	3D learning showed a statistically significant improvement in self-assessment of the frontal sinus drainage pathway (p=0.0266)	Some concerns
Kong (2015) [28]	RCT/n=61	Hepatic segment anatomy	3DV model OR 3DP physical model (3D structure)	Traditional (anatomical atlas)	Educational effectiveness (first and second examinations/knowledge retention)	Both 3DV and 3DP models were significantly better than the traditional anatomical atlas in the first and second examinations (p<0.05). Only the traditional method group had significant score declines between examinations	Some concerns
Kockro (2015) [9]	Randomised trial/n=169	Neuroanatomy (third ventricle)	Stereoscopic 3D VR lecture (dextrobeam system, stereoscopic projection, animated tour for large groups)	2D PowerPoint presentation (Standard screen, images/2D content from textbooks)	Examination results (MCQs), subjective evaluation	3D presentation was statistically non-inferior to traditional 2D presentation (p<0.0001). Students rated the 3D method superior in four domains (p<0.01)	Low risk
Ng (2015) [5]	RCT/n=72	Epitympanum anatomy (middle ear)	Interactive 3D computer model (Google Sketchup model viewed on iPad, enabling all-angle viewing)	Traditional (reading material and pictures/2D illustrations/textbook excerpts)	Examination performance (anatomy quiz/timed test), utility/subjective feedback	3D group mean score (65.1%) was significantly higher than the 2D group (32.4%) (p<0.001)	Low risk
Hopkins (2011) [27]	RCT/n=74	Muscles of mastication, skull structures	3D computer model OR hybrid (3D model + gross prosections)	Gross prosections and skulls	Knowledge test scores (pre- and post-test)	No significant effect of the instructional tool used on knowledge improvement (p=0.13)	Some concerns
Keedy (2011) [26]	Randomized, controlled study/n=46	Hepatobiliary anatomy	3D interactive multimedia module (computer-based, user-driven animations, virtual "fly-throughs")	Traditional textbook style (computer-based text document with static 2D images, content-matched)	Examination performance (adjusted post-test), satisfaction survey	No statistically significant difference in adjusted post-test scores (p=0.33). 3D group reported significantly higher overall satisfaction (4.5 vs. 3.7, p=0.02)	Some concerns
Tam (2010) [25]	RCT (cluster randomized)/n=34 attended exam	Gastro-intestinal anatomy	Computer program ("disect") (manipulating/reconstructing real CT images in 3D, self-directed or guided use)	Traditional (textbook, lecture notes, or anatomy atlas)	Knowledge of gastro-intestinal anatomy, attitudes	No difference in knowledge scores between guided use and self-directed use (p=0.52). Most students found the program easy to use and valuable	High risk
Nicholson (2006) [24]	RCT/n=57	Middle and inner ear anatomy (3D relationships)	Fully interactive 3D model (web-based tutorial, VRML file, arbitrary rotation, zoom, structure identification)	Web-based tutorial without a model (using text and 2D images)	Knowledge of 3D anatomical relationships	Intervention group mean score (83%) was significantly higher than the control group (65%) (p<0.001)	Low risk
Aridan (2024) [8]	Randomized trial/n=60	Neuroanatomy (white matter tracts of the cerebrum)	VR (HMD/headset, photorealistic 3D models, allows walking, exploration, and manipulation) or physical 3D printed models	Reading only (dissection manual with 2D images)	Theoretical/practical exam performance, learning experience	VR and physical models groups were significantly better than the Read-only group in questions requiring spatial understanding. VR learning experience rated higher than physical models	Low risk
Donnelly (2009) [23]	Experimental RCT (crossover design)/n=89	Cross-sectional abdominal anatomy	VHD software (simultaneously viewing 2D cross-sections and reconstructed 3D views)	Traditional dissection room materials (prosections and models)	Examination performance (cross-sectional anatomy identification)	No significant difference between the VHD group and the traditional methods group at any stage	Low risk
Bogomolova (2020) [15]	Double-center RCT/n=58	Lower limb musculoskeletal anatomy	Stereoscopic 3D AR model (HoloLens HMD, stereopsis, dynamic exploration, active user interaction) or Monoscopic 3D desktop model (2D screen, rotation, active user interaction)	2D anatomical atlas (Handouts from atlas/textbook, primarily 2D images)	Anatomical knowledge (factual, functional, spatial), visual-spatial abilities, and learning experience	Stereoscopic 3D AR group performed equally well as the 2D anatomical atlas group (p=1.00). The 2D anatomical atlas group outperformed the monoscopic 3D desktop group (p=0.042). Stereoscopic 3D AR facilitated knowledge acquisition in students with lower visual-spatial abilities	Low risk
Çeri (2021) [22]	Randomised study/n=120	Neuroanatomy, cardiovascular system, digestive system	3DG (digital 3D application)	MG or AG	Success scores/examination performance	3DG was significantly more successful in all anatomical subjects tested (neuroanatomy, cardiovascular system, digestive system) than both MG and AG (p<0.05 for all comparisons)	Some concerns
Eroğlu (2023) [21]	RCT (parallel-group)/n=87	Male genitalia anatomy (complex) and liver anatomy (less complex)	3D PDF (lecture videos included 3D PDFs/manipulated models, lecturer manipulates models for multiple angles/perspectives)	2D atlas (lecture videos included 2D atlas images, static)	Retention performance (immediate and delayed tests)	3D PDF was significantly better in the immediate test for complex anatomy (genitalia, p=0.017) but similar for less complex anatomy (liver, p>0.05)	Low risk
Yun (2024) [7]	Open-labeled crossover RCT/n=154	Human anatomy (heart) and Neuroanatomy (diencephalon/telencephalon/ventricles)	Virtual dissection (HMDs/life-sized touchscreen/tablets, manipulation, multi-angle observation, immersive, scenario-based content)	Traditional donor dissection (tutors guiding dissection/extraction/observation)	Academic performance (quizzes Q1, Q2), student satisfaction (esthetics, spatial ability, immersion)	Virtual group showed significantly higher mean Q1 quiz scores in neuroanatomy and in the human anatomy observation class (p<0.05). Satisfaction was significantly higher with all virtual devices than with donor dissection across various categories	Low risk
Du (2020) [6]	RCT/n=18	Unspecified anatomy (puzzles of the human body)	VR gaming system (HTC Vive, handheld controllers for moving/rotating models, SP or MP competition	Textbook reading (control group CG)	Multiple-choice test scores (knowledge retention), motivation	VR groups showed better memory retention than the CG group (the MP group scored significantly higher than the CG group on Day 12). The MP group had significantly higher stress levels than the SP group (p<0.001)	High risk

Impact on Student Performance in Anatomy Examinations

The evaluation of academic performance demonstrated that VT is generally non-inferior to traditional methods, but often superior when teaching structures that require explicit spatial comprehension or clinical correlation.

AR and MR: In the domain of knowledge acquisition, AR/MR demonstrated clear strength in specialized and complex clinical domains. An AR headset utilizing stereoscopic depth cues showed significantly superior performance in short-term recall for head and neck anatomy-radiology correlation compared to 2D screen visualization (p=0.022) [30]. In musculoskeletal anatomy, MR using a HoloLens achieved practical exam scores equivalent to those of traditional cadaveric dissection while requiring less time (3.6 hours versus 6 hours), indicating efficiency benefits [18]. Importantly, AR/MR proved beneficial for mitigating intrinsic learning difficulties: stereoscopic 3D AR models helped students with lower visual-spatial abilities achieve scores comparable to those of the 2D anatomical atlas group [15]. However, the MR HoloLens was found to be less effective than a textbook-style resource for learning interdisciplinary concepts related to lung and heart anatomy in one study (p=0.011), possibly due to distraction [19].

VR and simulators: VR, particularly when immersive or integrated with clinical simulation, excelled at complex spatial and functional anatomy.

Immersive VR demonstrated notable advantages in complex cardiac anatomy, yielding scores 23.9% higher overall than an independent study and showing a 26.4% improvement in visual-spatial content (p<0.001) [4]. A high-fidelity simulator-based course for coronary angiography also produced significantly higher global scores than traditional PPT courses (p<0.001), indicating improved clinical competency [11]. In terms of equivalence, immersive VR for neuroanatomy (ventricular system) was found to be as effective as online textbooks in post-test outcomes [10], and stereoscopic 3D VR lectures on the third ventricle were statistically non-inferior to 2D PowerPoint presentations (p<0.0001) [9]. Immersive VR was similarly shown to be as effective as cadaveric bone models for skeletal learning [12]. For virtual dissection, approaches using HMDs, touchscreens, and tablets resulted in significantly higher quiz scores in neuroanatomy and in observation classes for human anatomy (heart) compared with traditional donor dissection methods [7].

VI3DM: VI3DM demonstrated robust performance, especially when teaching intricate anatomical relationships.

For spatial concepts, fully interactive 3D ear models produced highly significantly higher scores than a 2D tutorial, particularly for knowledge of 3D anatomical relationships (p<0.001) [24], and interactive 3D computer models for middle ear anatomy similarly resulted in a significantly higher mean score (65.1%) compared with 2D illustrations (32.4%) (p<0.001) [5]. Regarding VI3DM benefits, VI3DM enhanced the conceptualization of external features and anatomical relations, yielding significantly greater score improvements than 2D photographs across five specimens [20]. Specifically, for the sacrum, VI3DM significantly improved conceptualization compared with 2D aids (p<0.005) [30]. In terms of complexity, 3D PDFs used in lecture videos were significantly more effective than 2D atlases for immediate tests of complex male genitalia anatomy (p=0.017). However, no difference was observed for less complex liver anatomy [21]. For equivalence, a 3D interactive multimedia module for hepatobiliary anatomy showed no statistically significant difference in adjusted post-test scores compared with a content-matched traditional textbook-style presentation (p=0.33) [26].

Impact on Knowledge Retention

Retention of knowledge was assessed over periods ranging from a few days to several months, yielding mixed results; however, some evidence suggested that interactive visualization helps mitigate the decline in knowledge scores over time.

AR and MR: The effectiveness of MR/AR in enhancing long-term retention remains uncertain. Veer et al. found no significant difference in knowledge retention scores between the MR HoloLens group and the textbook-style group assessed at two weeks [19]. However, in a study focusing on initial learners, year 1 students who learned complex anatomy (male genitalia) via 3D PDF videos performed significantly better on the delayed retention test administered 10 days later than the 2D atlas group (p=0.044) [21].

VR and simulators: VR interventions were frequently reported to show no statistical advantage in retention. Immersive VR for neuroanatomy showed no significant difference in anatomy knowledge during retention quizzes administered eight weeks post-intervention compared to the control group [10]. Similarly, VR used as a complementary tool showed no significant differences in short-, medium-, or long-term retention of anatomy content when compared to traditional methods alone [14]. An exception was found in a small VR gaming study, in which the multiplayer VR group showed better memory retention than the textbook-reading control group on day 126.

Virtual 3D models/visualization: Visualization tools demonstrated a potential advantage in slowing the rate of knowledge decay. When comparing a 3D visualization model (3DV) to an anatomical atlas for hepatic segment anatomy, it was found that the traditional method group was the only group that suffered a significant decline in scores between the initial and delayed examinations (p<0.05), indicating a benefit conferred by the 3DV model in retention [28].

Impact on Student Satisfaction and Engagement

Across all three modalities, subjective outcomes relating to motivation, engagement, and perceived spatial benefit were overwhelmingly superior for VT compared to traditional comparators.

AR and MR: AR/MR were rated highly for their ability to convey complex spatial information and increase motivation.

For spatial perception, participants rated the holographic model (HG) significantly higher than both the didactic lecture and the computer module for overall effectiveness, ability to convey spatial relationships, engagement, and motivation (all p<0.001), with 62% preferring the HG modality [16]. In terms of enjoyment and usefulness, the stereoscopic 3D AR group reported significantly greater enjoyment than the monoscopic 3D desktop and 2D atlas groups, and students consistently rated 3D display technologies, including stereoscopic 3D, highly for perceived effectiveness and ease of use [13]. Immersive VR and simulator-based environments also demonstrated strong motivational effects, often independent of measured knowledge gains. For motivation, immersive VR for neuroanatomy produced significantly higher scores on the instructional materials motivation survey (total score, attention, confidence, and satisfaction) compared with online textbooks (all p<0.01), with learners finding the VR experience significantly more engaging, enjoyable, and useful (p<0.01) [10]. For perceived spatial benefit, students greatly valued VR’s ability to support virtual “dissection and reassembly” of the heart, rating immersive VR as highly effective for teaching 3D orientation and anatomical relationships [12]. Additionally, virtual dissection devices consistently yielded significantly higher satisfaction than traditional donor dissection across categories such as esthetics, conceptual understanding, spatial ability, and immersion [7].

Virtual 3D models/visualization: General 3D viewing applications maintained a clear advantage in user satisfaction over 2D and text-based resources.

For satisfaction, the 3D interactive multimedia module for hepatobiliary anatomy received a significantly higher overall satisfaction rating than the traditional textbook-style approach (p=0.02) [26]. Regarding spatial clarity, participants using screen-based interactive 3D computer models for ear anatomy reported that the ability to manipulate the model made “spatial relations easier to understand” and enabled “better visualization of complex structures” [5], while the group using a 3D human anatomy application demonstrated higher success and satisfaction compared with those using anatomical models or atlases [22].

Discussion

This systematic review analyzed the effectiveness of various VT modalities, including VR, AR, and VI3DM, as complementary or alternative methods for teaching human anatomy to medical students, in comparison to traditional methods.

The objective results regarding academic performance were mixed, often showing parity with traditional methods, yet VT demonstrated superior effectiveness when the learning objective involved complex spatial relationships [5,11,20,24,30]. Structures such as the middle and inner ear [5,24], cardiac anatomy [4], and the sacrum [30] were learned significantly better using interactive 3D modalities, which reduce the cognitive load of mentally translating 2D images into 3D structures [9,26,27]. For instance, Nicholson et al. found that a fully interactive 3D model resulted in highly significantly higher scores for 3D anatomical relationships (p<0.001) compared to 2D images [24]. Similarly, VR/MR achieved equivalence with gold-standard methods, such as cadaveric dissection [18], often increasing efficiency by reducing the time required in the curriculum.

Crucially, the review identified an aptitude-treatment interaction: stereoscopic 3D displays (AR/VR) were particularly beneficial for students with lower visual-spatial abilities, helping them achieve knowledge gains comparable to high-ability students or those using traditional methods [15].

However, the most consistent finding across all technological modalities was the pronounced superiority of VT in enhancing subjective outcomes, specifically student satisfaction, motivation, engagement, and perceived spatial understanding [9,10,16,26]. Even when academic scores were statistically equivalent to those from traditional instruction (e.g., neuroanatomy VR vs. online textbook) [10], students reported a significantly better learning experience [9,10]. For example, holographic models were rated higher than didactic lectures and computer modules for overall effectiveness, spatial relationships, engagement, and motivation [16].

Comparison With Previous Reviews

The findings of this qualitative synthesis generally align with and elaborate upon the established body of quantitative meta-analyses concerning extended reality (XR) in anatomy education. While earlier systematic reviews reported no statistically significant differences in anatomy knowledge gains between XR interventions and traditional resources, more recent meta-analyses, which included a greater number of studies, have established a modest overall benefit. Specifically, García-Robles et al. found that XR technologies yield a medium positive effect on knowledge gains (standardized mean difference (SMD) = 0.40) compared to traditional approaches [31]. This benefit is most pronounced when XR is used as a supplemental/complementary learning resource (SMD = 0.52) and is significantly greater than for passive learning methods such as didactic lectures (SMD = 1.00) and textbooks/atlases (SMD = 0.32). The current review's key finding that VT is superior for complex spatial structures is consistent with Yammine and Violato’s meta-analysis [2], which demonstrated the educational superiority of 3DVT in the acquisition of spatial anatomy knowledge.

Despite the mixed nature of objective knowledge gains in our qualitative data, there is universal agreement on subjective outcomes. The consistent superiority of VT in promoting student satisfaction and enjoyment is strongly supported across all referenced systematic reviews. García-Robles et al. quantified this preference, reporting that 80% of all students who used XR devices found them helpful in learning anatomy [31]. Furthermore, the observation in this review that objective gains can be modest or context-dependent is supported by Wang et al. [32], who found that 3D visualization technology may struggle to improve test scores when regional biases are excluded, even though satisfaction and enjoyment remain significantly improved (SMD = 0.79).

Strengths

The primary strength of this review lies in its foundation of 27 RCTs, which strictly adhere to the rigorous criteria defined in the study protocol. This approach maximizes the internal validity of the findings [8,11]. The synthesis successfully segmented results by type and degree of interactivity (AR, VR, VI3DM), enabling nuanced conclusions [20]. The inclusion of studies that directly compared high-interactivity VT (MR/immersive VR) with high-fidelity traditional standards, such as cadaveric dissection, provides strong evidence of VT’s practical viability and efficiency gains [12,18]. Finally, the review identified the crucial aptitude-treatment interaction [15], demonstrating that stereoscopic depth cues specifically facilitate learning for students with lower visual-spatial abilities, an insight often missed in standard comparative studies.

Limitations

The review faced several methodological limitations, primarily stemming from the inherent challenges in educational technology research:

Significant heterogeneity was present across the included studies, encompassing differences in VT modality, anatomical focus, comparator groups, assessment methods, and learner level. Participants ranged from early medical students to senior or postgraduate trainees. This variability reflects the design of the primary trials and may limit the generalizability of findings across specific educational stages. This broad methodological and educational diversity necessitated a qualitative narrative synthesis, as the data could not be meaningfully pooled in a meta-analysis.

Risk of bias: Despite relying on RCTs, studies frequently suffered from unavoidable biases, such as the inability to blind participants to the intervention (e.g., comparing a headset to a textbook), which contributes to performance and detection biases ​​​​​and limits the generalizability of outcomes [10,16]. The high student satisfaction observed may be partly attributed to the novelty (Hawthorne) effect of the new technology [15].

Outcome gaps: Most studies focused on short-term recall and assessed it immediately after the intervention. Long-term retention data were scarce, inconsistent, or compromised by high attrition rates (leading to missing outcome data) in follow-up assessments [4,13,19,25]. Furthermore, reliance on 2D assessment tools (multiple-choice questions) may have inadvertently undermined the measured impact of VT by encouraging factual memorization over spatial comprehension [15,16,27].

Implications for Anatomy Education

The review supports the strategic integration of VT, not as a replacement, but as a supplementary resource in modern medical anatomy curricula, primarily leveraging its efficiency and motivational benefits.

Strategic deployment for spatial and complex topics: VT should be explicitly targeted to educational modules covering anatomical structures that require high cognitive and visuospatial skills. This strategic deployment is crucial because VT's superiority is confirmed in reducing the cognitive load required to understand complex 3D relationships [9]. Furthermore, institutions should utilize stereoscopic VT (AR/VR) to address the aptitude-treatment interaction, as this technology specifically facilitates learning for students with lower visual-spatial abilities [15].

Enhance efficiency and manage resources: The finding that methods like MR can achieve equivalent academic outcomes to cadaveric dissection while requiring less curricular time (3.6 hours vs. 6 hours) [9] validates VT as an efficient and resource-sparing tool for managing limitations in donor specimens and laboratory time [7].

## Conclusions

VT represents a decisive shift in anatomy pedagogy, offering unique solutions to the long-standing challenge of comprehending complex 3D structures amid dwindling traditional resources. Although VR and AR tools did not uniformly enhance factual recall compared with conventional methods, they achieved comparable learning efficacy, with demonstrated benefits for spatial understanding and efficiency, while consistently boosting student engagement and motivation. We conclude that advanced VT, particularly immersive and stereoscopic modalities, is an effective and highly desirable tool that merits inclusion in modern medical school curricula as a vital supplement and, potentially, an alternative to traditional teaching methods.
